# Diode Laser Frenectomy as a Safe and Effective Surgical Option for Lingual Ankyloglossia: A Case Report

**DOI:** 10.7759/cureus.71283

**Published:** 2024-10-11

**Authors:** Danielle Santos-Rodrigues, Luciane Hiramatsu Azevedo

**Affiliations:** 1 Postgraduate Program in Dentistry, Universidade de São Paulo, São Paulo, BRA

**Keywords:** ankyloglossia, frenotomy, lasers, lingual frenum, semiconductor laser

## Abstract

A high-power diode laser (808 nm) is a potentially cost-effective, effective, and safe option for lingual frenectomies. An eight-year-old female patient with mild difficulty in articulating some phonemes, Angle class III malocclusion with maxillary atresia, and bilateral anterior and posterior crossbite was indicated for diode laser frenectomy. The procedure was performed with the following parameters: λ = 808 nm (±20 nm), 1.5 W, in continuous mode. Energy delivery utilized a 300 μm-diameter quartz fiber maintained in contact with the irradiated tissue. A four-year follow-up revealed the preservation of tongue anatomy functionality, restored in conjunction with speech therapy. A high-power diode laser (808 nm) stands out as a prioritized alternative among available technologies for lingual frenectomy, particularly when administered by adequately trained professionals.

## Introduction

Ankyloglossia, defined as a congenital disease characterized by the shortening of the lingual frenulum or its atypical adhesion to the genioglossus muscle [1], has an average prevalence of 5% in the population [2]. The limitation of tongue mobility has deleterious local and systemic repercussions in all stages of life [2].

Ankyloglossia is associated with difficulties in breastfeeding, subsequent low weight gain in newborns, and early weaning, and this, in turn, can compromise child development [3]. In adulthood, ankyloglossia can trigger obstructive sleep apnea and cause atypical swallowing [4]. This, in conjunction with neuromuscular dysfunctions in elderly patients, favors aspirational pneumonia [5]. The most effective treatment for ankyloglossia is a surgical procedure [3]; however, apparently, there are excessive costs for procedures with dubious indications [6].

The risk of postoperative complications varies according to the age of the patients. Reports on complications are still lacking [7]; however, there is a potential risk of injury to the lingual caruncle and the anatomical structures of the tongue itself. Hemorrhages, pain, and postoperative infections are inherent in all surgeries [6]. In this scenario, laser frenectomy emerges as an alternative that reduces the probability of bleeding and the need for suturing the surgical wound; moreover, it reduces the operating time and the consumption of analgesics [8]. The aim of this case report was to highlight all the potential benefits of the correct use of high-power diode lasers for the surgical resolution of ankyloglossia, with a focus on favorable outcomes free of postoperative complications, the modulation of inflammation, and healing in young patients.

## Case presentation

The patient, an eight-year-old female, presented to the pediatric dentist with complaints of mild difficulty in articulating some phonemes and other speech disturbances. The patient's parents reported that the patient was undergoing speech therapy and otolaryngology follow-up and had received an indication for surgery, with which the family was hesitant to proceed, due to concerns about the intervention.

**Figure 1:**
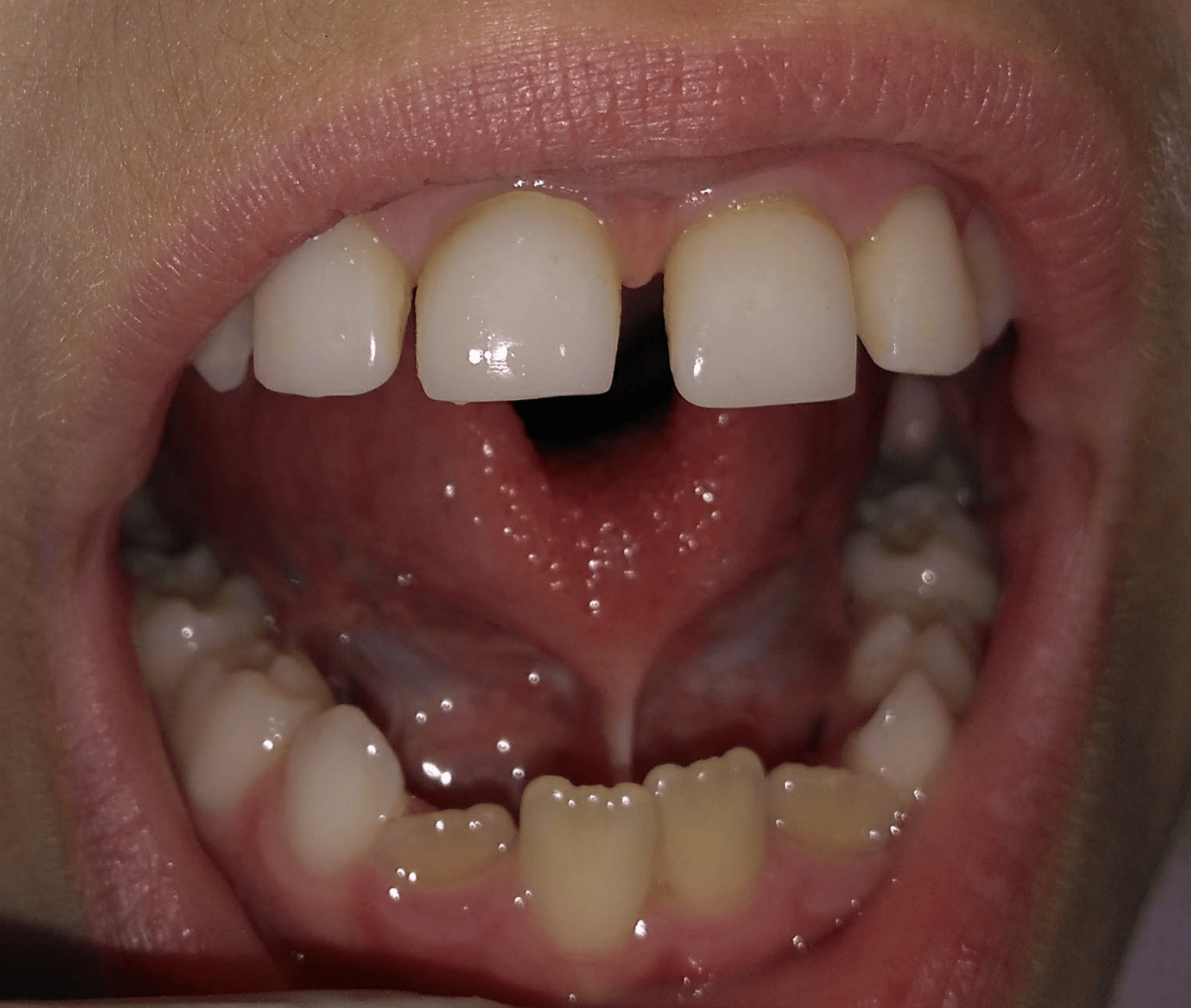
Preoperative tongue elevation

**Figure 2:**
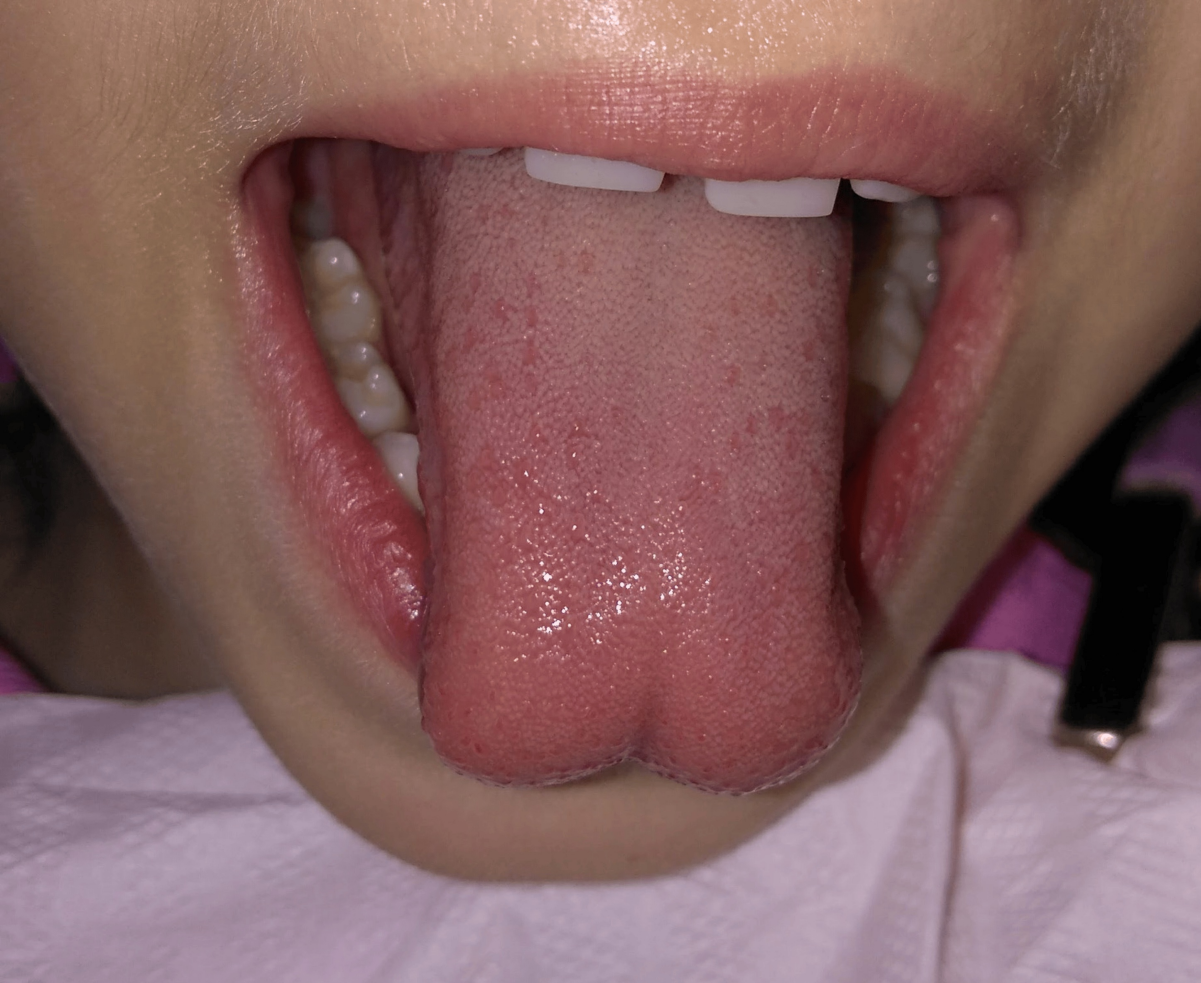
Preoperative tongue protrusion

Clinical examination revealed ankyloglossia, characterized by a short lingual frenulum that divided the tip of the tongue into two segments during tongue protrusion. Tongue mobility was limited (Figures 1, 2). Relative to the maxillomandibular relationship, the patient was classified as Angle class III, with maxillary atresia, in addition to bilateral anterior and posterior crossbite.

**Figure 3:**
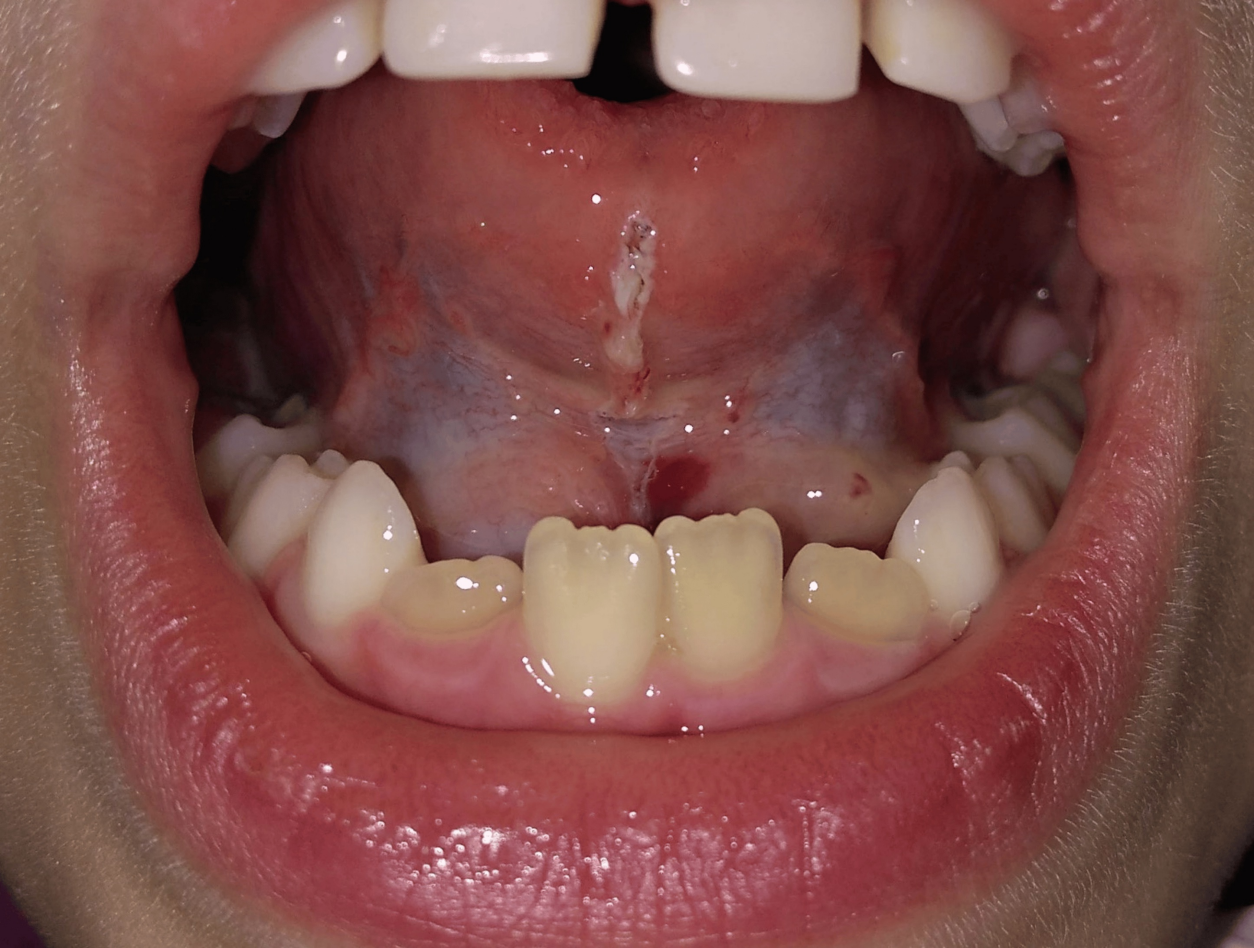
Immediate hemostasis postoperatively

The surgery was preceded by the anesthetic infiltration of the sublingual region with 2% lidocaine solution and 1:100,000 epinephrine. Frenectomy was performed using a high-power diode laser to incise the lingual frenulum and dissect its fibers (Thera Lase Surgery, DMC, São Carlos, Brazil) with the following parameters: λ = 808 nm (±20 nm), 1.5 W, in continuous mode. The energy was delivered by means of a 300 μm-diameter quartz fiber, previously activated with carbon paper and maintained in contact with the irradiated tissue. The procedure was performed without any complications. Immediate hemostasis was observed after making the incision, facilitating the visualization of the surgical field (Figure 3). The entire surgical procedure lasted no more than 10 minutes.

**Figure 4:**
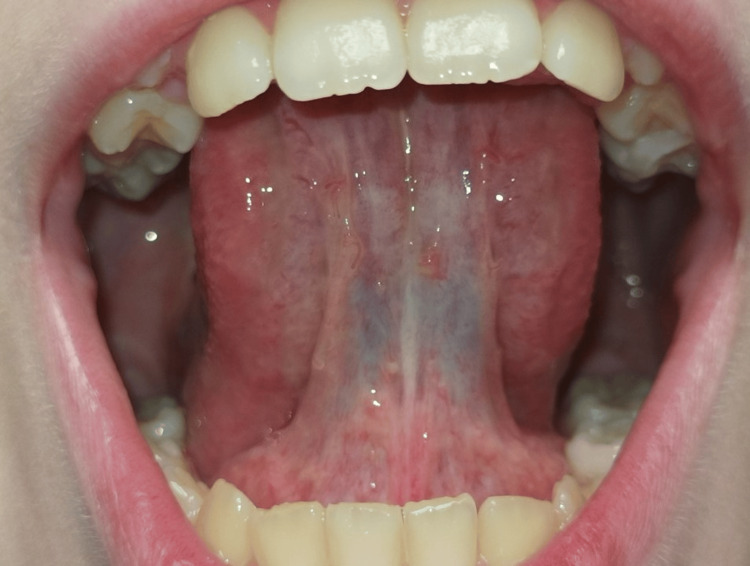
Follow-up after four years

The follow-up for this case continued for four years (Figure 4). During this period, the functions and anatomy of the tongue remained completely preserved. The patient regained tongue mobility and all related orofacial functions after speech therapy.

## Discussion

This case report presents a high-power diode laser (λ = 808 nm ± 20 nm) as a consistent and complication-free alternative to lingual frenectomy. The four-year follow-up of the patient confirmed the superiority of this technology in the healing process, favoring the re-architecture of the anatomical structures involved, which resulted in complete functional recovery. Other wavelengths, both for diodes and other modalities of light-emitting diodes, are known to be equally effective in surgically resolving ankyloglossia [8]. Lasers with a high affinity for water, such as carbon dioxide (CO_2_) and erbium lasers, do not achieve intraoperative hemostasis during incision. In contrast, the diode laser uses hemoglobin as its chromophore, facilitating the visualization of the surgical field throughout the procedure due to the absence of bleeding [9,10].

The specific advantage of diode lasers, such as the one used in the case reported, lies in the low cost of the acquisition of the device in the overall price per surgery. The literature has mentioned that the relevance of financial considerations in the indication of this procedure is as important as the clinical outcome itself [6]. With reduced surgical time, the cost of the clinical hour also decreased. Furthermore, the minimal surgical manipulation of tissues associated with the modulatory properties of the diode laser, immediate hemostasis, and the sterilization of the area of incision position it among the most appropriate technologies for lingual frenectomy in preventing complications [8]. The launched blue diode laser, due to its greater interaction with hemoglobin, which absorbs photothermal energy, may result in more effective vaporization than the same laser operating at infrared wavelength [11]. Other lasers, at various wavelengths, are also absorbed by hemoglobin, such as neodymium-doped yttrium aluminum garnet (Nd:YAG) or potassium-titanyl-phosphate (KTP) [12]. However, more comparative studies are needed to elucidate the real gain in the effectiveness of the use of such technologies [13].

However, it is essential to contextualize the results achieved by laser frenectomy, since they are only possible when the operator is fully qualified by additional specialized training. Potential thermal damage resulting from excessive energy poses irreversible risks in some cases [14]. The movement of the optical fiber must be continuous during the incision for the proper removal of muscle fibers. There is only one study reporting the occurrence of mucocele as a postoperative complication in laser surgery performed with unspecified dosimetric parameters [7].

## Conclusions

This case report suggests that high-power diode lasers (808 nm) are not only feasible but also a priority alternative among other available technologies for a lingual frenectomy. The specific properties of this type of laser favor a safe, fast, and effective procedure. Families and patients resistant to surgical recommendations tend to reconsider the procedure when informed about the unique advantages of diode lasers, both during and after the surgery.
